# Proficiency in performing radiofrequency ablation procedure for non-functioning benign thyroid nodules: a qualitative rather than quantitative matter

**DOI:** 10.3389/fendo.2024.1399912

**Published:** 2024-06-12

**Authors:** Spyridon Chytiris, Marsida Teliti, Laura Croce, Francesca Coperchini, Beatrice Grillini, Matteo Cerutti, Rodolfo Fonte, Flavia Magri, Mario Rotondi

**Affiliations:** ^1^ Unit of Endocrinology and Metabolism, Laboratory for Endocrine Disruptors, Istituti Clinici Scientifici Maugeri IRCCS, Pavia, Italy; ^2^ Department of Internal Medicine and Therapeutics, University of Pavia, Pavia, Italy

**Keywords:** thyroid nodules, benign, non-functioning, radiofrequency ablation, learning curve

## Abstract

**Objective:**

Radiofrequency ablation (RFA) is an emerging non-surgical treatment for benign thyroid nodules (BTN). Despite its proven safety profile, data on the learning curve (LC) required to achieve proficiency are still lacking.

**Materials and methods:**

The first 179 RFA procedures performed by a single operator in patients with non-functioning BTN were retrospectively analyzed. Six-month nodule volume reduction rate (VRR) ≥ 50% was regarded as reflection of proficiency. Multiple linear regression analysis has been performed to determine the relationship between the VRR and clinical variables. Cumulative sum (CUSUM) charts were plotted to assess LCs for all consecutive procedures and in relation to basal nodule size. In details, Group 1 (G1): 57 patients with small nodules (<10 ml); Group 2 (G2): 87 patients with intermediate nodules (10 – 25 ml); Group 3 (G3): 35 patients with large size (> 25 ml).

**Results:**

LC of all 179 procedures showed 3 phases: initial learning (1–39 procedures); consolidation (40–145 procedures); and experienced period (146–179 procedures). For G1 and G2 proficiency is achieved starting from the 10th procedure within the group (or 37th considering consecutively all procedures) and from the 59th procedure within the group (or 116th considering consecutively all procedures), respectively. LC of G3 did not detect operator proficiency.

**Conclusion:**

Specific LCs exist concerning the basal size of the nodule treated with RFA. In nodules with baseline volume > 25 ml suboptimal VRR has to be expected. Previously achieved experience on small-intermediate nodules does not seem to provide advantages in terms of higher VRR in the treatment of large nodules. Other potential and non-modifiable factors likely play a key role in the final volume reduction independently from the increased skill of the operator.

## Introduction

Thermal ablation for benign thyroid nodules is an effective and safe non-surgical treatment for benign thyroid nodules (BTN) (1, 2). Radiofrequency ablation (RFA), is a procedure performed through a trans-isthmic approach and a moving-shot technique, reported to display a high efficacy together with a low complication rate (3). In this regard, a recent interdisciplinary consensus statement of the American Thyroid Association aimed to provide a framework for the safe adoption and implementation of non-surgical ablation technologies for BTN, including data on the learning curve (LC) and necessary prior skillset (4).

LC is defined as the time taken and/or the number of cases required by an average operator to become proficient to be able to perform a procedure independently and obtaining a satisfactory outcome (5). The concept of the LC is often employed in surgery, where a constant stream of new skills must be acquired safely and effectively (6). Similarly, some Authors have addressed the issue of how many RFA procedures an operator may have to carry out before reaching a safe and competent level of performance.

Up to now, only three reports with an overall number of 291 patients have focused on LCs for thyroid RFA (7–9). Two of the three studies focused on the LC for a single operator (7, 8), and the other involved a team of two radiologists (9). According to these previous studies, physicians with prior expertise in thyroid ultrasound (US) and fine-needle aspiration (FNA) biopsy may have to carry out at least 20–30 RFA procedures before reaching a safe and competent level of performance. Although these previous experiences have provided a first and meaningful insight into the role of the operator experience on clinical efficacy in RFA for benign nodules, some additional aspects require further investigation. Just to give an example, the baseline nodule volume was consistently reported to be related to RFA treatment outcomes (10, 11), but the role of basal volume of the treated nodule was not fully characterized when LC is considered.

Thus, the primary outcome of this study will be to assess the LC for thyroid RFA of a single operator in treating non-functioning BTN, using volume reduction rate (VRR) as a proficiency marker. The secondary goal will be to identify both baseline patient-related as well as technical aspects of the ablation procedure potentially related to the therapeutic outcome as assessed by VRR.

### Patients and methods

This cohort study enrolled the first 179 consecutive patients with non-functioning BTN receiving RFA treatment at the Endocrinology and Metabolism Unit of the ICS Maugeri, Pavia. The reasons for performing RFA included: 1) subjective compressive symptoms such as difficulty in swallowing or the feeling of local pressure and/or cosmetic concerns; 2) increase in the volume of the nodules over an ultrasound follow-up in the last years; 3) patient’s preference for RFA rather than surgery. Inclusion criteria were: 1) serum TSH, FT4, and FT3 levels within the normal range; (3) at least one benign cytological result at fine-needle aspiration (FNA). All patients signed an informed consent concerning the future use of their clinic data for research purposes and data collected remained strictly confidential and anonymous, according to the ethical rules of our Hospital institutions and to the Declaration of Helsinki.

### Procedure

All patients referred for RFA treatment underwent a complete thyroid work-up, including a measurement of serum FT4, FT3, and TSH. A baseline US of the neck was performed by the same endocrinologist (S.C) who performed the RFA procedures. The thyroid nodule’s location, volume, and characteristics were collected, and all nodules were classified based on EU-TIRADS criteria (12). Thyroid nodule volume was calculated using the ellipsoid volume formula as follows: volume = 0.525 × length × width × depth. US-guided FNA was performed at least once in all patients, and cytological results were provided according to the Italian Thyroid Cytology Classification System 2014 (13). The procedures were performed in a sterile setting. Local anesthesia with Lidocaine 2% was administered at the skin puncture site and the perithyroidal space. No hydro dissection or anesthetic infusion was made in the peri- or under-capsular layer. The operator (S.C.), an endocrinologist with more than two decades of expertise in thyroid imaging, fine-needle aspirations, core-needle biopsies, and percutaneous ethanol therapy, initiated RFA treatment with the first patient enrolled. All RFA procedures were performed using a 18-gauge internally cooled electrode, 7 or 10 cm length with a 10 mm active tip (RF AMICA PROBE, Hs Hospital Service S.p.A, Italy). The US-guided moving-shot technique with a trans-isthmic approach was applied (14). During the procedure, patients remained in a supine position with mild neck extension. They were advised to report pain, and voice testing was performed at regular intervals. The starting level of energy delivered per second was 30 W. The energy output was progressively increased if no response was visible on the US within 60 seconds. At the end of the procedure, the patient applied mild compression of the treated thyroid lobe for 20 min. If necessary, an icepack was applied to relieve pain. After 2 h of post-procedural observation, a neck US was performed. Standard follow-up included outpatient visits with FT4, FT3, and TSH after 6 and 12 months. Nodule volumes were measured by the US after 1, 6, and 12 months. Nodal volume reduction was expressed as volume reduction ratio, calculated as follows: VRR = ((initial volume − final volume)/(initial volume)) × 100. Technique efficacy was defined as a volumetric reduction ≥50% of the initial nodule volume.

### Data analysis

The statistical analyses were performed using the SPSS software (SPSS, Inc., Evanston, IL) and JMP (version 17, SAS Institute Inc., Cary, NC).

The LCs of the technical efficacy were analyzed using the calculated cumulative sum (CUSUM), a graphics-based analysis approach typically used for monitoring change detection. The CUSUM of the deviations of each sample value from the target value is plotted in the y-axis and the case number is plotted on the x-axis. The target value is set to VRR 50%. The cumulative sum of the deviations upper and below the target value is indicated by the black dots. The upper and lower control limits are determined as ± 20 standard deviations (SD) from the target value and are drawn as red horizontal lines. Results are reported as mean values with S.D., medians with ranges, or as proportions. A multiple linear regression model was constructed by entering VRR as a dependent variable while chronological order, age, nodule composition, nodule location, nodule baseline volume, and applied energy served as covariates. A *p*-value <0.05 was considered statistically significant.

## Results

### Basic characteristics of all patients

Throughout the study-span, 179 benign thyroid nodules (178 patients) were treated by RFA. In one patient, two separate nodules were treated. The mean age of the patients was 58,10 ± 13,17 years (range, 23 to 92). The mean baseline nodule volume was 17,73 ± 13.29 ml (range, 2.39 to 80.58). In all, 80 were solid-predominantly solid; 92 were mixed; and 5 were cystic-predominantly cystic. At 6 -month post-RFA the mean VRR of 179 nodules was 58,38 ± 17.28% and volumetric reduction ≥50% of the initial nodule volume was achieved in 78.13% of patients. The treatments were performed by using a mean total estimated energy of 19337,80 ± 11517,67 J and a mean energy per volume of 1361,86 ± 883,28 J/ml. **Table 1** shows the patient’s demographic and clinical characteristics.

**Table 1:** Patient’s demographic and clinical characteristics.

Age (years)	58,10 ± 13,17
Male/Female (n°)	28/151
TSH (mUI/mL)	1,96 ± 4.18
Nodule location	
Right Isthmus Left	86 3 90
Nodule composition	
Solid- predominantly solid Mixed Cystic-Predominantly cystic	82 92 5
Nodule baseline volume (ml)	17,73 ± 13,29
Depth (mm)	23,84 ± 6,27
Width (mm)	30,37 ± 8,50
Length (mm)	40,77 ± 10,23
VRR at 6 mo (%)	58,38 ± 17.28
VRR ≥ 50% at 6-month (yes/no)	131/48
RFA time (sec)	643,12 ± 308,3
Applied energy (J)	19337,80 ± 11517,67
Applied energy (J/ml)	1361,86 ± 883,28

Results are expressed as mean ± standard deviation or numbers. TSH, thyroid-stimulating hormone; VRR, volume reduction rate; RFA, radiofrequency ablation.

### Learning curve analyses

**Figure 1:**
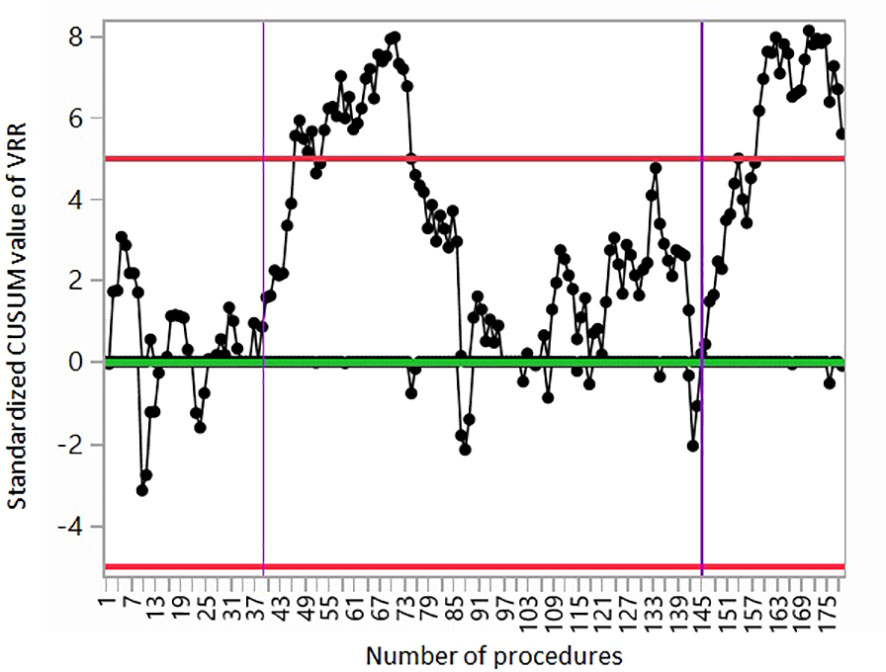
CUSUM analysis of the LC of the 179 RFA procedures. The CUSUM of the deviations of each sample value from the target value of VRR 50% is plotted in the y-axis. The case number plotted on the x-axis shows consecutively all 179 procedures. VRR, volume reduction rate.

Based on the CUSUM analysis of the 179 consecutive procedures, changes in the slope could be observed after 39 and 145 procedures, allowing to divide the LC into 3 distinct phases. Indeed, the vertical line on the CUSUM Chart indicates that a positive shift in the VRR values started around sample 39 (end of the initial LC), “plateaus” between cases 40–145 with higher variability in the CUSUM trend (consolidation phase), subsequently a new shift is recorded from case 146 (proficiency phase). **Figure 1** shows the CUSUM analysis of the LC of the 179 RFA procedures.

To identify baseline and procedural parameters that correlate with VRR values, a multiple linear regression analysis was performed including chronological order, age, nodule composition, nodule location, nodule baseline volume, and applied energy (**Table 2**). The only independent predictor of 6-month VRR was nodule baseline volume (β = -0,31, 95% confidence interval = -0,501, -0,123, *p* = 0,001).

**Table 2:** Multivariate analysis of factors predicting VRR.

Independent variables	Coefficient (β)	Lower limit 95% CI	Upper limit 95% CI	*p-value*
Chronological order	0,01	-0,14	0,16	0,899
Age (years)	-0,088	-0,241	0,066	0,262
Nodule location	0,064	-0,078	0,207	0,372
Nodule composition	0,035	-0,109	0,179	0,632
Nodule baseline volume (ml)	-0,31	-0,501	-0,123	0,001
Applied energy (J)	-0,002	-0,18	0,176	0,981

VRR, volume reduction rate, CI, confidence interval.

Based on the above results, to explore the possibility that differences in nodule baseline volume might influence proficiency, patients were stratified into three groups according to nodule volume. In detail, Group I encompassed 57 patients with small nodules (<10 ml); Group 2 encompassed 87 patients with intermediate nodules (volume 10 – 25 ml); Group 3 encompassed 35 patients with large size (volume > 25 ml). As a further step, a single CUSUM chart for each group was drawn.

**Figure 2:**
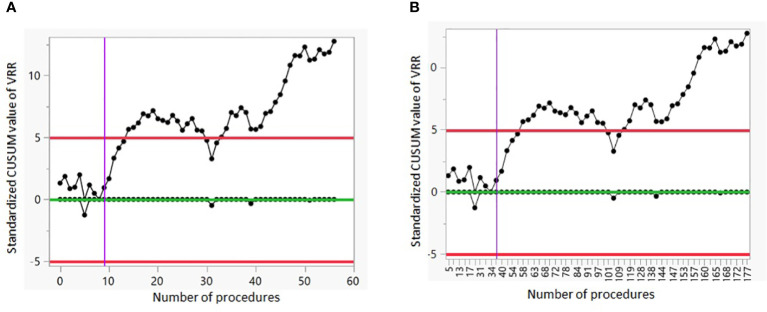
CUSUM analysis of the LC of G1. **(A)** The CUSUM of the deviations of each sample value from the target value of VRR 50% is plotted in the y-axis. The case number plotted on the x-axis shows the 57 procedures performed on patients with small nodules. **(B)** The CUSUM of the deviations of each sample value from the target value of VRR 50% is plotted in the y-axis. The case number plotted on the x-axis shows consecutively all 179 procedures. VRR, volume reduction rate.

**Figures 2A, B** show the CUSUM analysis of the LC of G1. The vertical line on the CUSUM Chart indicates that a positive shift in the VRR values started around sample 10 when taking into account only the patients with small nodules; and “moves” to sample 37 when we consider consecutively all patients treated.

**Figure 3:**
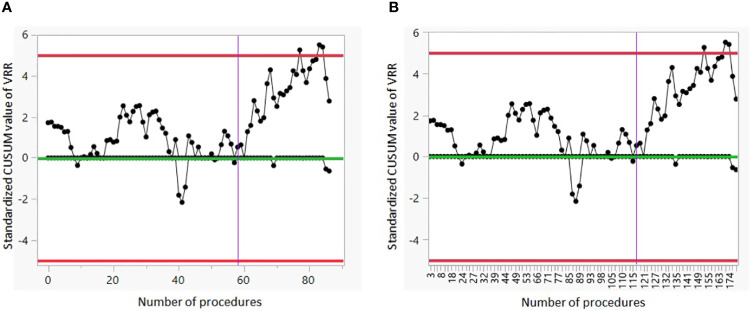
CUSUM analysis of the LC of G2. **(A)** The CUSUM of the deviations of each sample value from the target value of VRR 50% is plotted in the y-axis. The case number plotted on the x-axis shows the 87 procedures performed on patients with intermediate nodules. **(B)** The CUSUM of the deviations of each sample value from the target value of VRR 50% is plotted in the y-axis. The case number plotted on the x-axis shows consecutively all 179 procedures. VRR, volume reduction rate.

**Figures 3A, B** show the CUSUM analysis of the LC of G2. The vertical line on the CUSUM Chart indicates that a positive shift in the VRR values started around sample 59 when taking into account only the patients with intermediate nodules; and moves to sample 116 when we consider consecutively all patients treated.

**Figure 4:**
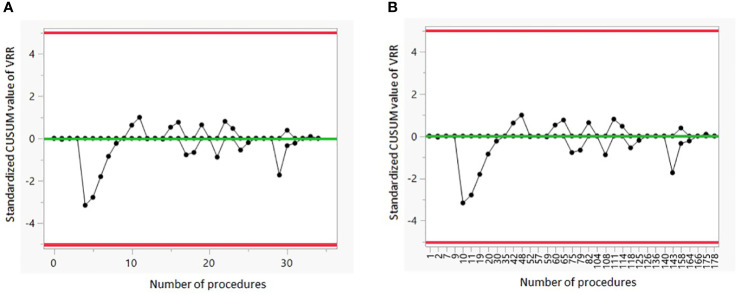
CUSUM analysis of the LC of G3. **(A)** The CUSUM of the deviations of each sample value from the target value of VRR 50% is plotted in the y-axis. The case number plotted on the x-axis shows the 35 procedures performed on patients with large nodules. **(B)** The CUSUM of the deviations of each sample value from the target value of VRR 50% is plotted in the y-axis. The case number plotted on the x-axis shows consecutively all 179 procedures. VRR, volume reduction rate.

**Figures 4A, B** show the CUSUM analysis of the LC of G3. There is no positive or negative shift in the VRR values.

## Discussion

The present study follows some previous ones, clearly indicating that a 6-month VRR of 50% represents a realistic and rapidly achieved goal in most patients treated by RFA for benign thyroid nodules. According to a large body of evidence, demonstrating that RFA-associated complications are rather rare, the main outcome measure to determine RFA proficiency has become the rate of volume reduction (15). Thus, three previous studies have conventionally focused on a minimum threshold for procedural volume in order to achieve a higher 6-month VRR (7–9). Bom et al. demonstrated that about 40 procedures are required to achieve a VRR >50% and of 79% in the following 63 patients (9). Russ et al, in their experience with the first 90 patients treated, showed that a LC existed regarding technical efficacy, VRR, and ablation ratio, a relatively new objective parameter to assess the percentage of the successfully ablated nodule. However, after 60 procedures, improvement only persisted in the latter and for small to medium nodules. Recently, Kuo et al, using the CUSUM chart, identified two inflection points of the LC at the 20th and 65th cases, respectively (8).

Thus, a certain degree of amelioration in the skilled operator was reported after a minimum of ablation procedures ranging from 40 to 60. However, it is rather disappointing to find that, although a rather limited number of procedures is sufficient to achieve a VRR >50% in most patients, further increasing the numbers of procedures performed, leads to limited additional increase in VRR. This appears in agreement with the findings by De Andrea et al. who, in an international prospective randomized controlled trial, compared the volume reduction obtained in patients treated in two centers (Italy and Korea) with different experience of the moving-shot technique (respectively 50 vs 3000 procedures). The results in terms of volume reduction were impressive in the treated group, both in Italy and Korea, with a slightly greater efficacy in the latter group where experience in the moving-shot technique is consolidated, but without a significant difference between the two countries. To note, the baseline volume was larger in the Italian series (16.4 ± 2.5 mL vs. 13.9 ± 3.3 mL, p=0.009) (16).

The results of our study demonstrate that single-operator LC patterns in achieving higher 6-month VRR are profoundly different according to the baseline volume of the nodule treated. Indeed, as assessed by CUSUM charts, it is evident that while for small volume nodules (< 10 ml) a relevant increase in VRR is observed starting from the 10th procedure, for intermediate volume nodules (10–25 ml), ∼60 RFA procedures were required to obtain a comparable amelioration in the VRR. Consistently, when treating large volume nodules (> 25 ml), even when performed by an operator who has simultaneously demonstrated optimal volume reduction rate in the treatment of lower volume nodules, ∼ 35 previous procedures do not warrant a mean 6-month VRR > 50%. Furthermore, according to our result, it could be stated that an experienced proceduralist (∼180 procedures performed) may fail to achieve a 6-month VRR > 50% when large nodules are taken into account.

The above evidence would support the concept that specific LCs exist concerning the size of the nodule treated and, more importantly and surprisingly, that previously achieved experience (as assessed by the number of total procedures performed) on small-intermediate nodules does not provide advantages (at least in terms of higher VRR) when the same operator faces large nodules.

Although the here reported LCs outcome in relation to baseline thyroid volume may result from a lower efficacy and efficiency of the moving-shot technique on large nodules, other potentially relevant variables should be taken into account and discussed.

Beyond the operator’s mastery, the patient’s characteristics (i.e. age and ability to maintain neck hyperextension for increasing amount of time) and nodule’s characteristics (large thyroid nodules and particular anatomical site and/or surrounding critical structures) may all contribute to the final outcome when assessed by the degree of VRR (17).

The above considerations suggest a further aspect that might be important to highlight which is related to the desired therapeutic goal. Indeed, in treating nodules with small and medium sizes, it is desirable to achieve the highest possible volume reduction. In patients with large nodule sizes, we should look at RFA as a technique providing personalized treatment, in that, we may actually fix a given end-point for each patient. Thus, it is reasonable to assume that the final aim of the procedure is to provide relief of symptoms which is not always observed in parallel with greater VRR. In line with this statement, Papini et al. reported that a 50% volume reduction is most often sufficient to improve patients’ symptoms (18).

The current problem is that we should distinguish between the technical efficacy of RFA (i.e. VRR) and the clinical efficacy of RFA (i.e. therapeutic success).

Our data suggest that in nodules with baseline volume > 25 ml suboptimal VRR has to be expected and the concept of LC may not be applicable. Potential and non-modifiable factors such as nodule vascularization, composition, anatomical site and biological characteristics as well as patients’ compliance likely play a key role in the final volume reduction independently from the increased skill of the operator. Consistent with this, Bernardi et al, investigated the outcomes of re-treatment on symptomatic BTN with a VRR <50% after the initial thermal ablation treatment (19). The RFA re-treatment led to greater VRR as compared to the first treatment only in small and medium nodules (<30 mL) but not in large nodules (>30 mL).

The current study has several strengths. The cohort size is roughly double compared to other studies on LC, increasing our results’ representativeness and accuracy. Secondly, the cases included were consecutive and inclusive of all RFA procedures performed by the operator, unraveling a realistic picture of daily clinical practice. Thirdly we used a specific statistical approach for LC evaluation (CUSUM analysis).

Potential limitations of the present study include the single-center and the retrospective design, which inherently restricts external generalizability, even though the here reported results are in overall agreement with those provided by previous studies. Furthermore, this study lacks an evaluation of the initial ablation ratio (IAR), calculated as the ratio between the ablated volume and the total nodule volume before RFA (20). Its potential value as a parameter to document operator skills was recently demonstrated by Russ et al., showing that a single-operator LC for achieving an IAR >90% was substantially longer than required for a VRR >50% and exceeded 90 procedures when large nodes are involved (7). However, the accuracy of IAR for long-term volume reduction and regrowth prediction remains to be established (21, 22).

In conclusion, the results of the present study demonstrate that: i) RFA is a safe and rapidly acquired procedure at least for small to medium size nodules; ii) the therapeutic efficacy of RFA should not be exclusively assessed by VRR as relevant clinical benefits may be obtained also for sub-optimal VRR; iii) RFA treatment should be regarded as an additional therapeutic tool for personalized treatment of patients bearing thyroid nodule.

Future prospective longitudinal studies, specifically aimed to evaluate the long-term outcome as well as the role of 6 month VRR to predict final size of the nodules will be required to further characterize the relationship between technical efficacy and clinical efficacy of the RFA procedure.

## Data availability statement

The raw data supporting the conclusions of this article will be made available by the authors, without undue reservation.

## Ethics statement

Ethical approval was not required for the studies involving humans because Formal approval by the ethical committee was not required in accordance with the Italian regulation for non-interventional (observational) retrospective studies concerning human beings (AIFA Guidelines for Observational Studies, see www.AIFA.gov). The studies were conducted in accordance with the local legislation and institutional requirements. The participants provided their written informed consent to participate in this study.

## Author contributions

SC: Conceptualization, Data curation, Writing – original draft. MT: Writing – review & editing, Data curation, Writing – original draft. LC: Writing – review & editing. FC: Writing – review & editing. BG: Data curation, Writing – review & editing. MC: Data curation, Writing – review & editing. RF: Writing – review & editing. FM: Writing – review & editing. MR: Conceptualization, Writing – review & editing.
